# Mapping regulators of cell fate determination: Approaches and challenges

**DOI:** 10.1063/5.0004611

**Published:** 2020-07-01

**Authors:** Aditya Kumar, Prashant Mali

**Affiliations:** Department of Bioengineering, University of California, San Diego, La Jolla, California 92093, USA

## Abstract

Given the limited regenerative capacities of most organs, strategies are needed to efficiently generate large numbers of parenchymal cells capable of integration into the diseased organ. Although it was initially thought that terminally differentiated cells lacked the ability to transdifferentiate, it has since been shown that cellular reprogramming of stromal cells to parenchymal cells through direct lineage conversion holds great potential for the replacement of post-mitotic parenchymal cells lost to disease. To this end, an assortment of genetic, chemical, and mechanical cues have been identified to reprogram cells to different lineages both *in vitro* and *in vivo*. However, some key challenges persist that limit broader applications of reprogramming technologies. These include: (1) low reprogramming efficiencies; (2) incomplete functional maturation of derived cells; and (3) difficulty in determining the typically multi-factor combinatorial recipes required for successful transdifferentiation. To improve efficiency by comprehensively identifying factors that regulate cell fate, large scale genetic and chemical screening methods have thus been utilized. Here, we provide an overview of the underlying concept of cell reprogramming as well as the rationale, considerations, and limitations of high throughput screening methods. We next follow with a summary of unique hits that have been identified by high throughput screens to induce reprogramming to various parenchymal lineages. Finally, we discuss future directions of applying this technology toward human disease biology via disease modeling, drug screening, and regenerative medicine.

## I. INTRODUCTION

During development, cells become increasingly specialized to a terminally differentiated state. This highly regulated process is controlled in part by the expression of transcription factors (TFs) that form specific network modules to ensure stable gene expression and cell identity.^1,2^ Maintaining cellular identity is critical for healthy organ function, leading some to theorize that terminally differentiated cells lack the ability to transdifferentiate to different lineages.^3^ However, it has since been shown that the overexpression of only a few TFs can change cell identity.^4–6^ For example, although there are estimated to be over 1500 different human TFs,^7^ the overexpression of a single TF is capable of reprogramming fibroblasts into myoblasts.^8^ Moreover, alternative combinations of TFs or other means of reprogramming, such as chemical and environmental cues, can lead to the same cell type, suggesting that the genetic regulatory networks characteristic of a specific cell type may be established by different reprogramming factors.^9–11^ As such, enormous effort has been applied to identify these master regulators of cell fate. This concept has since attracted enormous interest in the regenerative medicine field as a means to regenerate diseased tissue by reprogramming stromal cells into post-mitotic parenchymal cells, yielding promising results across multiple lineages *in vitro* and even improving outcomes in *in vivo* models.^12–14^

However, low reprogramming efficiencies with known factors limit clinical translation.^15^ Although only a few TFs are needed to induce reprogramming, the large set of potential combinations of TFs makes finding the optimal reprogramming cocktail for each cell type daunting.^16^ Original screening methods were labor intensive and low throughput, but the recent application of high throughput genetic and chemical screening approaches has systematically identified new factors to improve efficiency.^17–20^ In this review, we will discuss screening strategies utilized to reprogram cells toward parenchymal lineages. We will address considerations in interpreting results from screens while highlighting the strengths and weaknesses of different approaches. We will then highlight key factors found through screening approaches before concluding by discussing future directions of applying this technology toward human disease biology via disease modeling, drug screening, and regenerative medicine.

## II. RATIONALE, CONSIDERATIONS, AND LIMITATIONS FOR USING HIGH THROUGHPUT SCREENS TO IDENTIFY REPROGRAMMING FACTORS

### A. Rationale for utilizing high throughput screens to identify reprogramming factors

Although original strategies focused on the overexpression of a single TF to reprogram cells, it quickly became evident that specific combinations of multiple TFs are needed for efficient reprogramming of most cell types.^21,22^ As a result, a trial and error approach was adopted where a pool of TFs thought to be important for lineage specification was delivered to cells and then a single TF was removed; if reprogramming efficiency went down, the factor was reinstated but if the efficiency went up or remained unchanged the factor was removed until a minimum combination was achieved.^23,24^ A transformative success utilizing this approach was the reprogramming of fibroblasts into induced pluripotent stem cells (iPSCs) via overexpression of Oct4, Sox2, c-Myc, and Klf4.^25^ 24 different genes associated with maintenance of embryonic stem cell identity were first overexpressed followed by removal of genes until the identification of the four factors needed to generate iPSCs.^25^ This approach was rapidly adopted within the scientific community and applied to reprogram stromal cells into parenchymal cells of various lineages, including cardiomyocytes,^26^ neurons,^27,28^ hepatocytes,^29^ and pancreatic islet cells.^30,31^ Additionally, further work built upon this strategy by screening other genetic factors, such as microRNAs and chromatin modification,^32–35^ as well as chemical reprogramming via the addition of cytokines and small molecule inhibitors.^36–40^ While these pioneering studies definitively demonstrated the ability to directly reprogram stromal cells into parenchymal cells, this trial and error approach is limiting in the number of combinations that could be tested, resulting in low reprogramming efficiency.^41,42^ As such, computational frameworks were developed to predict TF combinations that could directly reprogram one cell type into another.^43^ While useful, these frameworks are based upon a similar concept of identifying TFs that are differentially expressed between cell types and serve more as a starting point in identifying potential combinations.

**FIG. 1.**
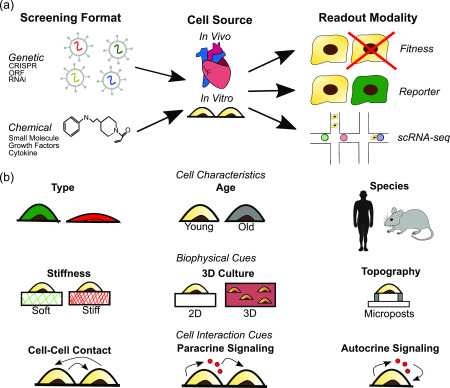
Overview of the components and considerations involved in high throughput screening for the identification of reprogramming factors. (a) Listed are the common modalities utilized for reprogramming screens. (b) In addition to understanding the strengths and weakness of screen components, additional consideration of how cell characteristics, biophysical cues, and cell interaction cues can influence results is critical to properly interpret results from screens.

Given the potential number of TF combinations that are possible, high throughput screening approaches are critical for the unbiased identification of optimal TF combination. Recent technological advances have allowed researchers to move past the trial and error approach and instead combined high throughput genetic or chemical perturbations with phenotypic or transcriptomic readouts to identify factors governing cell fate (Table I).^16–19,35,44–61^ These screens are either performed in arrayed format, where perturbations are maintained in separate culture conditions, or pooled format, where perturbations are assayed *en masse*. Although chemical screens must be run in an arrayed format, genetic screens are moving toward a pooled format. Both formats have their strengths and weakness. Pooled formats are easy to run and allow for comprehensive profiling of large libraries but require next generation sequencing (NGS) to map perturbations to phenotype and require single cell readout modalities. However, advances in NGS technology now make readout of pooled screen results quick and cheap, fueling the widespread use of pooled screens at a rapid pace. Arrayed formats require specialized automation to perform large screens, but it is very easy to correlate perturbations to phenotypes and to perform more rigorous phenotypic assays. Development of technology such as liquid handling robots and high content imaging allows for larger arrayed screens, but application is still limited due to the cost of these setups (Table II). Regardless of how the screen is performed, they all rely on three key components: the screening format, the cell type/source, and the readout modality [Fig. 1(a)]. Consideration of the strengths and weakness of different methods for these components is critical to interpret results from high throughput screens and extract meaningful data.

**TABLE I.** Examples of reprogramming factors identified using high throughput screening.

Starting cell	Ending cell	Screen type	Readout	Unique hit	References
Mouse epiblast stem cell	iPSC	CRISPRa	Oct4-GFP	Sall1	19
Mouse embryonic fibroblast	iPSC	ORF overexpression	Nanog-GFP	Glis1	44
Mouse embryonic fibroblast	iPSC	ORF overexpression	Tra-1–60+	Hhex, Hlx	45
Mouse embryonic fibroblasts	iPSC	RNAi	Oct4-GFP	Trim28	46
Mouse embryonic fibroblast	iPSC	RNAi	Alkaline phosphatase+	Tox4	47
Mouse embryonic fibroblast	iPSC	Chemical	Oct4-GFP	AM580+EPZ004777+SGC0946 + 5-aza-2-deoxycitidine	48
Human dermal fibroblast	iPSC	Chemical	Alkaline phosphatase+	SAHA-PIP Ì	49
Mouse embryonic stem cell	Neuron	CRISPRa	Tubb3-CD8	Ezh2	18
Human fibroblasts	Neuron	ORF overexpression	scRNA-seq	Pax6+Neurog2+Dlx2+Zic1, Pax6+Neurog2 +Dlx1+Isl1	50
Mouse embryonic fibroblasts	Neuron	ORF overexpression	Tau-GFP	Brn3c+Ascl1	51
Mouse embryonic skin fibroblast	Neuron	Chemical	Tau-GFP	Forskolin+ISX9+ CHIR99021+I-BET151	52
Human fetal lung fibroblasts	Neuron	Chemical	Map2+	Kenpaullone+Prostaglandin E2+Forskolin+BML210+ Aminoresveratolsulfat+PP2	53
Mouse cardiac fibroblast	Cardiac progenitor cell	CRISPR knockout	Nkx2-5-GFP	Dmap1	54
Mouse cardiac fibroblasts	Cardiomyocytes	ORF overexpression	αMHC-GFP	Znf281	55
Mouse cardiac fibroblasts	Cardiomyocytes	ORF overexpression	GCAMP+	Hand2+Nkx2.5+Gata4 +Mef2c+Tbx5	56
Mouse tail-tip fibroblasts	Cardiomyocytes	RNAi	αMHC-GFP	Bmi1	35
Mouse cardiac fibroblast	Cardiomyocytes	RNAi	αMHC-GFP	Zrsr2	57
Human cardiac fibroblasts	Cardiomyocytes	Chemical	Cardiac troponin T-GFP	SB431542+XAV939	58
Human iPSC	Endothelial cell	ORF overexpression	Fitness+ scRNA-seq	Etv2	17
Mouse embryonic fibroblast	Epicardial cell	ORF overexpression	scRNA-seq	Atf3+Gata6+Hand2	16
Mouse embryonic fibroblasts	Dendritic cell	ORF overexpression	Clec9a-tdTomato	Pu.1+Irf8+Batf3	59
Mouse embryonic stem cell	Primoridal germ cell	CRISPR knockout	Stella-GFP + Esg1-tdTomato	Nr5a2, Zfp296	60
Mouse embryonic stem cell	2C-like cell	CRISPRa	scRNA-seq	Dppa2, Smarca5	61

**TABLE II.** Overview of various screening platforms.

	Equipment needed	Strengths	Weaknesses
CRISPR	1. Flow cytometer for reporter readout	1. Allows for endogenous gene overexpression	1. Off target risk, albeit small
	2. Next generation sequencers	2. Easy to develop large-scale library	2. Gene regulation not as strong as ORF systems
ORF	1. Flow cytometer for reporter readout	1. Strong overexpression	1. Difficult to make into large-scale library
	2. Next generation sequencers	2. Allows for expression of specific isoforms	2. Knockdown studies not readily feasible (require dominant negative versions of target protein coupled with strong overexpression)
RNAi	1. Flow cytometer for reporter readout	1. Allows for knockdown of critical genes	1. High off target risk impacts signal to noise in the screens
	2. Next generation sequencers	2. Easy to develop large-scale library	
Chemical	1. High content imaging system	1. Assay impact across a range of doses.	1. High throughput screening requires automation
	2. Liquid handling station	2. Easy to interpret results	2. Repeated dosing of small molecules

### B. Considerations in interpreting results from high throughput screens

#### 1. Genetic and chemical screening formats

The two most common genetic screening formats performed are open reading frame (ORF) overexpression and clustered regularly interspaced short palindromic repeats (CRISPR) screens. Although ORF overexpression and CRISPR screens have similar goals, e.g., genetic perturbation, differences in how they modify gene expression lead to different strengths and weaknesses.^62^ We will also briefly discuss RNA interference (RNAi) technology, which has played an important role in reprogramming screens^63^ but has been largely phased out in favor of CRISPR screens.^64,65^ Alternatively, chemical agents that target specific pathways or induce epigenetic remodeling can also serve as a means for cellular reprogramming. Several groups have performed high throughput chemical screens as an alternative or to augment genetic screens.^66^ We will discuss the strengths and weakness of the various screening formats below as well as summarize these points in Table II.

ORF overexpression has classically been utilized to perform reprogramming studies.^6,67^ This method involves cloning the entire coding sequence of a TF into a plasmid controlled by a promoter to drive exogenous gene expression. The plasmid is then packaged in a viral vector and delivered to the host cells so that the gene can be transcribed. The use of a promoter results in TFs being stably and dramatically overexpressed.^68^ As would be expected, the degree of overexpression appears to be key to induce reprogramming; high overexpression of certain genes has a profound impact on the reprogramming efficiencies to iPSCs, where ten to twenty-fold increase in Oct4 and Klf4 gene expression led to higher quality fully reprogrammed iPSCs,^69^ and cardiomyocytes, where a twofold additional increase in Mef2c overexpression resulted in a tenfold increase in reprogramming efficiency.^70^ Furthermore, by defining the coding sequence, this approach allows for the expression of specific isoforms or mutant forms of a gene. This is important as specific isoforms may be needed to induce reprogramming.^71,72^ The main disadvantage of ORF overexpression is the difficulty in scaling up libraries to perform a screen due to the large size of ORFs.^68^ However, publicly available genome-scale ORFeome collections have been developed that contain validated sequences mapping to over 13 000 genes and can be rapidly swapped into a desired vector.^73^

The CRISPR/Cas9 complex was originally adapted to allow for the efficient deletion or insertion of DNA sequences into the genome. The complex is directed to its target site by a short single guide RNA (sgRNA) sequence that recognizes and binds to a specific DNA sequence in the genome. The Cas9 nuclease is then able to cut the DNA, preventing gene expression.^74,75^ CRISPR knockout strategies have been used to reprogram cells via the inhibition of gene expression of TFs critical for lineage specification. For example, deletion of MyoD in C2C12 myoblasts led to transdifferentiation to adipogenic cells.^76^ More recently, a catalytically dead form of Cas9 (dCas9) has been developed that can be fused to transcriptional agonists or antagonists to increase or decrease gene expression endogenously.^77–80^ The main advantage of this approach is the ability to modulate gene expression without permanently modifying the genome. CRISPR activation (CRISPRa) induces gene expression through the fusion of dCas9 to transcriptional activators, such as VP64-p65-Rta, synergistic activation mediators, and SunTag, that have a DNA binding domain and a domain to activate transcription.^78–80^ On the other hand, CRISPR interference (CRISPRi) suppresses gene expression through the fusion of dCas9 to transcriptional repressor peptides, such as KRAB, that sterically block transcriptional initiation or elongation.^81^ CRISPR screens are popular due to the relative ease in developing large sgRNA libraries due to their small size, allowing for potentially genome wide perturbations.^82^ The library size depends upon the target gene list, with multiple sgRNAs targeting each gene (typically 3–10). Correspondingly, genome-scale CRISPR screens have libraries ranging from 60 000 to 200 000 elements.^83^ To modulate gene expression, sgRNAs are designed to target the promoter region or transcriptional start site of the gene.^84,85^ Care must also be taken that sgRNAs are specific for their target sequence. Furthermore, some genes are controlled by multiple enhancers, which may result in inefficient gene activation or repression.^86,87^

In this regard, ORF overexpression approaches allow for strong overexpression of genes as well as the expression of specific isoforms or mutants, while CRISPRa allows for endogenous gene expression to more physiological levels and ease of use given that generating sgRNA libraries is much easier than ORF libraries. As such, these two approaches should be seen as complementary; indeed, a previous study comparing CRISPRa and ORF screens for resistance to MEK inhibitors noted that both screens shared a number of top hits while also identifying unique ones.^62^ Given these factors, it is important to recognize the strengths and weaknesses when performing CRISPR or ORF overexpression screens.

For loss of function (LOF) studies, RNAi via the introduction of short-hairpin RNAs (shRNA) was commonly performed in the reprogramming field. Whereas ORF and CRISPR strategies target genomic DNA, RNAi promotes the degradation of complementary targeted mRNA. As such, RNAi results in a knockdown of gene expression but not a complete knockout.^65^ This feature is useful when assessing developmentally critical genes, as often is performed in reprogramming screens, where a complete knockout could be lethal. In addition, the ease of creating a shRNA library made LOF screens an attractive alternative to ORF overexpression screens. Genome wide RNAi libraries can contain a similar number of elements to CRISPR libraries.^88^ However, off-target effects are high as shRNAs can recognize and degrade mRNAs with imperfect complementary sequences,^89^ potentially modulating expression of many transcripts and inducing phenotypes that can be dominant over intended behavior.^90^ In a study that compared the on and off target effects of sgRNAs and shRNAs targeting the same genes, it was shown that 97.4% of sgRNAs had larger on target than off target effects, whereas only 41.8% of shRNAs had the same effect.^91^ As a result, CRISPR-Cas based screening has become an important approach in the field. The development of CRISPRi, which enables precision knockdown,^92^ has also contributed to the growing toolset of complementary approaches for enabling functional genetic screening.

In addition to genetic screens, chemical screens that regulate cell signaling pathways or the activity of histone and DNA modifying enzymes have gained interest as an alternative or to supplement genetic screens in reprogramming studies.^93,94^ The main advantage of a chemical approach is that it does not require the use of viral vectors, which reduces the risk of genomic instability or mutations. Furthermore, chemical compounds are more readily administered compared to the use of viral vectors and thus also more readily translatable.^93,94^ Similar to genetic screens, chemical screens were first utilized to reprogram fibroblasts into iPSCs. Mouse embryonic fibroblasts were reprogrammed into iPSCs using a purely chemical cocktail composed of valproic acid, CHIR99021, RepSox, tranycypromine, forskolin, 3-deazaneplanocin A, and arotinoid acid.^95^ This quickly led to the development of chemical cocktails capable of reprogramming stromal cells into various parenchymal cells. Chemical reprogramming shows clinical promise as well as it has recently been shown that *in vivo* reprogramming of fibroblasts to cardiomyocytes post-myocardial infarction is possible with a purely chemical cocktail, albeit involving the use of seven compounds.^96^ Thus, similar to genetic reprogramming, the major issues associated with chemical reprogramming involve identifying compounds that are important for reprogramming while limiting the number of compounds necessary. However, high throughput chemical screening is much more labor intensive than genetic screening as chemical screens must be performed in an arrayed format as described earlier.^58,93,97^ Furthermore, there are concerns of off-target effects unless better targeting chemical compounds can be produced.^98^ Nonetheless, chemical screens offer an important method for cell reprogramming while further informing genetic screening targets by providing a better understanding of the signaling pathways involved in reprogramming.

#### 2. Readout modalities

Regardless of how the screen is performed, a phenotypic or transcriptomic readout is required to identify reprogrammed cells. The most common readout modalities utilized are fitness, reporter, or single cell RNA sequencing (scRNA-seq) readout.

Fitness readouts involve the enrichment or depletion of screening factors based on viability changes at the end of the screen.^86,99^ To begin, cells are perturbed with the screen followed by additional culture to allow for the effects of perturbation to take place. Afterwards, genomic DNA is isolated from the remaining cell population and sequenced via NGS to map barcodes back to the starting library. Computational frameworks are used to determine differences in relative abundance of perturbations at the beginning and end of the screen to identify important factors. Top hits are then validated individually or in smaller screens and additional biological assays can then be performed to understand the mechanism by which enrichment or depletion occurs.^82,100^ While these screens are simple to run, their application is limited as many cellular processes do not always involve changes in cellular viability, such as cell reprogramming.^101,102^ However, fitness screens have been performed in the context of identifying factors responsible for the maintenance of stem cell or progenitor cell self-renewal^54,103^ or the reprogramming of stem cells into parenchymal cells.^17^ In this context, TFs that dropped out of the screen were investigated as potential reprogramming factors owing to the proliferation disadvantage associated with undergoing reprogramming without division.^17^

Reporter readouts, which involve the activation of a marker unique to a specific cell lineage, have classically been used to identify reprogrammed cell populations.^18,26,104^ Generally, a fluorescent reporter gene is driven by a cell type specific promoter to allow for the selection of reprogrammed cells via fluorescence activated cell sorting (FACS) after completion of the screen.^101^ Common markers include green fluorescent protein under the control of alpha myosin heavy chain (αMHC) promoter for cardiomyocytes or beta-tubulin for neurons.^18,26^ This readout is most commonly used for chemical screening due to its dependence on high content imaging to identify factors.^94,105^ Although this readout modality has great applicability to reprogramming studies, there are some drawbacks. A unique marker only expressed by the reprogrammed cell must be identified, which may be difficult for every cell type. Furthermore, expression of the marker does not provide information on the global cellular transcriptome, thus limiting the ability to distinguish partially reprogrammed cells from fully reprogrammed ones.^106^ For example, although a similar number of cells were αMHC positive for fibroblasts from different origins transduced with Gata4, Mef2c, and Tbx5, there were differences in the number of cells positive for cardiac troponin T between the groups.^26^ Similar issues are associated with stem cell reprogramming, where partially reprogrammed clones may be positive for Nanog but not Oct4.^107^ As such, any hit requires additional validation to separate false positives from confirmed factors.

scRNA-seq readouts involve the use of readable gRNAs or expressed barcodes that allow for mapping of phenotype changes to specific gene perturbations.^108,109^ Whereas the previous screening approaches can assay for only simple phenotypes, such as viability or marker expression, scRNA-seq allows for screening of more complicated phenotypes and allows for a better understanding of the perturbation consequences. Several techniques, such as Perturb-Seq,^110^ Crop-Seq,^111^ CRISP-Seq,^112^ and Mosaic-Seq,^113^ have been developed that combine pooled barcoded CRISPR perturbations with scRNA-seq to assess the effects of single and multiple perturbations on the cell signaling response. In the reprogramming field, scRNA-seq has been utilized to understand transcriptomic differences that account for reprogramming heterogeneity, identifying barriers found in resistant cells that can be removed with additional factors.^114–117^ Thus, scRNA-seq not only has utility as a readout but also as a tool to parse out the global effects of perturbation. Recently, this technology has been applied to high throughput reprogramming screens. Barcoded ORF overexpression libraries have been utilized to identify TFs responsible for reprogramming to endothelial^17^ and epicardial-like^16^ states based on their transcriptomic profiles. By combining TF overexpression with scRNA-seq, transcriptomic consequence of perturbations can be assessed, which allows for the identification of cell types as well as the separation of fully and partially reprogrammed cells. To successfully detect hits, the perturbation must have a strong effect or many cells carrying the specific perturbation must be sampled. Given the high sample preparation cost, profiling enough cells can be difficult. Furthermore, there are issues in separating runs due to sample-dependent batch effects.^114,118,119^ However, there is significant interest in improving these weaknesses given the wealth of information gained through scRNA-seq. Improved computational techniques to reduce batch effects^120,121^ and false positives^122,123^ as well as techniques to allow for higher throughput through scRNA-seq multiplexing^124,125^ have already been developed to improve data quality and reduce cost. Additional strategies, such as coupling scRNA-seq with other reporters to only sequence reprogrammed cells,^17^ have been utilized to reduce cost and improve the confidence of scRNA-seq data.

It should be noted that the duration of screens is context dependent, in that the timing is dependent on the phenotype of interest and the time it takes for the genetic perturbation to be established and the resulting effect to manifest. In addition, duration is also readout dependent. Generally, *in vitro* assays involving fitness readouts take 2–4 weeks to allow for the enrichment of the population. Assays with reporter or scRNA-seq readouts are generally quicker than those with fitness readouts as they do not require population enrichment.^126^

#### 3. Dependence on environmental conditions and starting cell populations

In addition to the components associated with the screen, it is important to understand how environmental conditions and starting cell populations can influence the results of a screen [Fig. 1(b)]. For example, it has been noted that *in vivo* reprogramming is both more efficient and induces cells with greater maturity than *in vitro* reprogramming with the same factors.^127^ One explanation for this phenomenon is biophysical and biochemical differences between *in vivo* and *in vitro* extracellular environments.^128,129^ Dynamic changes in the extracellular matrix occur during disease,^130,131^ and numerous papers have demonstrated how mimicking these changing properties can induce changes in cell behavior.^132–134^ These conditions are dramatically different from those in traditional reprogramming screens, in which cells are grown in plastic petri dishes. Topographical cues, such as microgrooves and micrograted substrates, have been shown to improve reprogramming of fibroblasts to iPSCs,^135^ neurons,^136^ and cardiomyocytes^137^ via epigenetic modulation. However, responses to other cues appear to be cell type specific. Whereas matrix stiffness improves reprogramming efficiency to iPSCs and influences stem cell lineage specification, it had no effect on reprogramming to cardiomyocytes.^137–139^ Instead, culture in 3D fibrin hydrogels improved cardiomyocyte reprogramming.^140^ Biochemical cues via cell-cell contacts and paracrine or autocrine signaling have also been shown to influence reprogramming efficiency. For example, co-culture of stromal cells with the parenchymal cell of interest improves reprogramming efficiency, viability, and functionality.^141,142^ Secreted factors can also influence reprogramming efficiency. It has been shown that blocking autocrine vascular endothelial growth factor signaling via knocking down vascular endothelial growth factor receptor 2 promotes stem cell self-renewal and somatic cell reprogramming.^143^ Thus, it is important to note that possible transcriptomic changes driven by cell and environmental interactions rather than reprogramming factors can increase variability within the screen. This point is especially important in interpreting scRNA-seq data where the local environment of each cell is dependent on its interactions with neighboring cells.

In addition to environmental conditions, it is important to note that reprogramming cocktails are heavily dependent upon the starting cell population age, source, and species. Studies comparing reprogramming efficiencies of fibroblasts to iPSCs from young (2 months old) and old mice (>2 years old) found that the older cells exhibited a twofold reduction in reprogramming efficiency.^144^ Although this trend is not quite as clear in human cells, with high variance of reprogramming efficiency of fibroblasts to iPSCs from 12 individuals between the ages of 8–64 years but not correlated with age,^145^ it has been noted that iPSCs generated from older patients have an increased number of mutations,^146^ though this does not necessarily alter their differentiation capability.^147^ Cell source is an additional consideration that is shown to influence reprogramming responses to the same factors. For example, Gata4, Mef2c, and Tbx5 overexpression was most efficient for mouse cardiac fibroblast reprogramming to cardiomyocytes^26^ but a separate study using mouse embryonic fibroblasts found that myocardin, Mef2c, and Tbx5 was the best combination and the addition of Gata4 was detrimental.^148^ Reprogramming efficiencies using the same factors are also different for different cell sources.^26,149^ These results are perhaps not surprising given that cells from different sources will have different epigenetic profiles that will alter their response to reprogramming factors. However, fibroblasts within the same organ are a heterogeneous population with different embryonic origins,^150,151^ suggesting that even cells from the same source may respond differently to reprogramming factors. Finally, species-specific differences between mouse and human cells must be taken into consideration. Gata4, Mef2c, and Tbx5 overexpression is insufficient to reprogram human cardiac fibroblasts. Instead, the addition of Esrrg, Mesp1, myocardin, and Zfpm2 was needed for sarcomere formation, calcium transients, and action potentials.^58^ Furthermore, whereas reprogramming of human cells to cardiomyocytes occurred anywhere from 4 to 11 weeks after transfection, mouse cell reprogrammed within two weeks.^26,152^ This trend holds for other cell types as it is generally noted that human cells reprogram slower and with lower efficiency than mouse cells.^6^ Given these results, reprogramming cocktails must be validated in human cells to be considered viable targets for regenerative medicine.

### C. Limitations of high throughput screens

In Secs. II B 1 and II B 2, we have highlighted various screening methods and modalities and identified weakness associated with each one. However, there are additional limitations associated with all screens that must be considered.

First, it is difficult to recapitulate multilineage differentiations seen in normal development. Whereas stem cell differentiations often produce both the parenchymal and supporting cell populations,^153^ genetic or chemical perturbations only produce a single or few cell types. As a result, additional cocktails must be discovered for each cell type. As mentioned above, co-culture can play an important role in reprogramming efficiency but is also important for cell maturation. For example, spontaneous neuronal activity and bursting behavior only occurs when reprogrammed neurons are co-cultured with astrocytes.^154^

Second, screens require a large number of cells to ensure good coverage of the library. To confidently separate true reprogramming hits from false positives, multiple cells must receive each combination of reprogramming factors. Given that combinations scale exponentially, screens must be designed in a manner that finds the balance between the number of factors and combinations in each cell.^126,155^ This can be an issue when using primary cells with limited proliferative capacity or are difficult to transduce. Thus, a common approach is to use immortalized cells for the initial screen followed by primary cells for targeted screens.^126^ There are obvious issues with this strategy given the high chance for false positive and negatives. In this context, ORF overexpression generally needs fewer cells per perturbation as it drives stronger phenotypic effects compared to CRISPR overexpression.^17^ Regardless, it is important to consider how many factors and combinations are feasible given the starting cell population.

Third, reprogrammed cells must remain committed to the new lineage once the perturbation is removed to have any relevance in an *in vivo* setting where constant dosing is not possible. Thus, the endogenous cellular network must be rewired and stably reprogrammed. As a result, it is important to monitor cells over time to ensure no signs of de-differentiation. For example, previous studies have noted that reprogrammed cardiomyocytes can lose sarcomere formation and marker expression after a month in culture in serum-containing media but maintained identity when cultured in defined conditions.^156^ Better understanding of the mechanism leading to loss of identity could potentially lead to the identification of factors that improve lineage commitment.

## III. APPLICATIONS OF CELL REPROGRAMMING TO TISSUE ENGINEERING AND REGENERATIVE MEDICINE

Given the care needed to set up, execute, and interpret results from large-scale screens, it is important to understand the strengths and weakness of the different applications of cell reprogramming. There are numerous interesting biological questions associated with understanding the regulators of cell fate and also important applications of reprogramming technologies to tissue engineering and regenerative medicine. As such, researchers have applied reprogrammed cells for disease modeling or the replacement of diseased parenchymal tissue with varying success.

Similar to iPSC-differentiated progenies, directly reprogrammed cells have been utilized in tissue engineering applications. Tissue engineered blood vessels have been produced via reprogrammed endothelial and smooth muscle cells, resulting in increased survival of mice after transplantation of the graft containing both cell types compared to transplantation with only the decellularized vessel.^157^ Additional applications of reprogrammed cells include drug screening, where induced hepatocytes exhibited similar drug metabolizing response and comparable toxicity prediction ability to primary human hepatocytes for 25 different compounds.^158^ Finally, patient specific reprogrammed cells have also been created that faithfully recapitulate phenotypes associated with the disease. For example, reprogrammed neurons from a patient with Huntington's disease exhibited neuritic breakdown, abnormal neuritic branching, increased cell death, and aggregation of mutant huntingtin.^159^ Similar recapitulation of deficits in neuron behavior associated with other neuronal diseases has also been demonstrated.^160^ The biggest issue with utilizing directly reprogrammed cells for tissue engineering applications is the risk of exhausting the original cell population. Cells must be immortalized to ensure self-renewal, risking alteration in reprogrammed cell behavior. The generation of iPSCs, which are inherently self-renewing, does not run this risk. Furthermore, the application of reprogrammed cells to tissue engineering is heavily dependent on the reprogramming efficiency of the cells. Whereas iPSC differentiation efficiency to cardiomyocytes is routinely greater than 90% after two weeks,^161^ reprogramming efficiencies are at best 50% after four weeks and involve a complicated assortment of genetic and chemical factors.^58^ Additional research is needed to identify factors that improve efficiency to allow for applications of reprogrammed cells to tissue engineering.

Applications of reprogrammed cells for regenerative medicine are already ongoing and appear very promising. *In vivo* reprogramming has been reported to ameliorate the effects of diseases such as stroke,^14^ diabetes,^13^ and myocardial infarction.^12^ There are many advantages that make direct reprogramming an attractive approach for regenerating tissue. Reprogrammed cells integrate into the organ,^12–14^ an important consideration given persistent issues with survival and engraftment with cell transplantation strategies.^162^ In addition, direct reprogramming is cheaper, safer, and faster than stem cell differentiation as it skips the pluripotent and intermediate progenitor stages.^15^ For example, *in vivo* reprogramming occurs within two months for most cell types, which is often the time needed to simply establish a stable iPSC line.^12–14^ Finally, the overexpression of specific genes allows for control of final cell fate. For example, specific subtypes of certain cells, such as excitatory or inhibitory neurons, can be generated via the overexpression of different TFs.^163,164^ However, there are limitations associated with direct reprogramming that must be overcome to translate this technology to the clinic. The use of viral vectors to deliver reprogramming factors carries the risk for insertional mutagenesis.^165^ To reduce this risk, viral vectors that do not integrate into the host genome has been utilized. One of the more attractive options is delivery via adeno-associated virus (AAV) as it does not integrate into the genome, has a low immunogenicity profile and high transduction efficiency and specificity based on AAV capsid serotype, and is used already in the clinic.^166^ Furthermore, additional research is attempted to further improve the therapeutic capacity of AAV vectors through the engineering of novel AAV capsid constructs. For example, recombinant AAV vectors have improved specificity and transduction efficiency, reducing the viral load needed for gene delivery.^167^ Additionally, improvement of reprogramming efficiency will further reduce the time needed to produce enough reprogrammed cells to have a clinical benefit. Given reduced efficiency in reprogramming human cells *in vitro*,^6,26,152^ it is likely that this trend will hold *in vivo*. Thus, improving delivery methods and identifying factors that improve efficiency are necessary for clinical translation of reprogramming technology.

## IV. SUMMARY AND FUTURE OUTLOOK

Direct reprogramming has rapidly emerged as a powerful tool for generating post-mitotic parenchymal cells lost to disease. Although numerous genetic, chemical, and environmental cues have been identified to induce reprogramming, the ideal cocktails for each cell type remain elusive. To systematically identify new factors, high throughput genetic and chemical screening approaches have been utilized. These approaches have greatly aided our understanding of genetic interactions associated with cell fate and identified new factors responsible for reprogramming. Given the great promise already seen in direct reprogramming to regenerative medicine, improving reprogramming efficiency by the identification of better factors will aid in the translation of this technology to the clinic.

## AUTHOR CONTRIBUTIONS

A.K. and P.M. conceived and wrote the manuscript.

## Data Availability

Data sharing is not applicable to this article as no new data were created or analyzed in this study.
